# Supplementary material for: Resolving chaperone-assisted protein folding on the ribosome at the peptide level
Source: Nat Struct Mol Biol. 2024 Jul 10;31(12):1888–97. doi: 10.1038/s41594-024-01355-x (PMC11638072; doi:10.1038/s41594-024-01355-x)
Supplement: Supplementary file 1 — Supplementary Table 1 [file 41594_2024_1355_MOESM1_ESM.pdf]

nature structural & molecular biology

Supplementary information https://doi.org/10.1038/s41594-024-01355-x

Resolving chaperone-assisted protein folding on the ribosome at the peptide level

In the format provided by the authors and unedited

Name	DHFR length (aa)	Amino acid sequence
**1-37[RNC]**	1-37	MSKGEELFTGVVPILVELDGDVNGHKFSVRGEGEGDATNGKLTLKFICTTGKLPVPWPTLVTTLTYGVL<br>CFSRYPDHMKRHDFFKSAMPEGYVQERTISFKDDGTYKTRAEVKFEGDTLVNRIELKGIDFKEDGNILG<br>HKLEYNFNSHNVYITADKQKNGIKAYFKIRHNVEDGSVQLADHYQQNTPIGDGPVLLPDNHYLSTQSVL<br>SKDPNEKRDHMVLLEDVTAAGITHGMDELYK**GSGS**LEVLFQGP**GSGSGSGSGSGSGSGSGSGSGSGSGS**<br>**ENLYFQGSG**MISLIAALAVDRVIGMENAMPWNLPADLAWFKRNTLNWWWPRIRGPPGS
**1-64[RNC]**	1-64	MSKGEELFTGVVPILVELDGDVNGHKFSVRGEGEGDATNGKLTLKFICTTGKLPVPWPTLVTTLTYGVL<br>CFSRYPDHMKRHDFFKSAMPEGYVQERTISFKDDGTYKTRAEVKFEGDTLVNRIELKGIDFKEDGNILG<br>HKLEYNFNSHNVYITADKQKNGIKAYFKIRHNVEDGSVQLADHYQQNTPIGDGPVLLPDNHYLSTQSVL<br>SKDPNEKRDHMVLLEDVTAAGITHGMDELYK**GSGS**LEVLFQGP**GSGS**MISLIAALAVDRVIGMENAMPW<br>NLPADLAWFKRNTLNKPVIMGRHTWESIGRPLPGRKNIILSSWWWPRIRGPPGS
**1-106[RNC]**	1-106	MSKGEELFTGVVPILVELDGDVNGHKFSVRGEGEGDATNGKLTLKFICTTGKLPVPWPTLVTTLTYGVL<br>CFSRYPDHMKRHDFFKSAMPEGYVQERTISFKDDGTYKTRAEVKFEGDTLVNRIELKGIDFKEDGNILG<br>HKLEYNFNSHNVYITADKQKNGIKAYFKIRHNVEDGSVQLADHYQQNTPIGDGPVLLPDNHYLSTQSVL<br>SKDPNEKRDHMVLLEDVTAAGITHGMDELYK**GSGS**LEVLFQGP**GSGS**MISLIAALAVDRVIGMENAMPW<br>NLPADLAWFKRNTLNKPVIMGRHTWESIGRPLPGRKNIILSSQPGTDDRVTWVKSVDEAIAACGDVPEI<br>MVIGGGRVYEQFLPKWWWPRIRGPPGS
**1-126[RNC]**	1-126	MSKGEELFTGVVPILVELDGDVNGHKFSVRGEGEGDATNGKLTLKFICTTGKLPVPWPTLVTTLTYGVL<br>CFSRYPDHMKRHDFFKSAMPEGYVQERTISFKDDGTYKTRAEVKFEGDTLVNRIELKGIDFKEDGNILG<br>HKLEYNFNSHNVYITADKQKNGIKAYFKIRHNVEDGSVQLADHYQQNTPIGDGPVLLPDNHYLSTQSVL<br>SKDPNEKRDHMVLLEDVTAAGITHGMDELYK**GSGS**LEVLFQGP**GSGS**MISLIAALAVDRVIGMENAMPW<br>NLPADLAWFKRNTLNKPVIMGRHTWESIGRPLPGRKNIILSSQPGTDDRVTWVKSVDEAIAACGDVPEI<br>MVIGGGRVYEQFLPKAQKLYLTHIDAEVEGDTHFPWWWPRIRGPPGS
**1-126[RNC]<br>mutant<br>(V40A, V75H, I91L)**	1-126	MSKGEELFTGVVPILVELDGDVNGHKFSVRGEGEGDATNGKLTLKFICTTGKLPVPWPTLVTTLTYGVL<br>CFSRYPDHMKRHDFFKSAMPEGYVQERTISFKDDGTYKTRAEVKFEGDTLVNRIELKGIDFKEDGNILG<br>HKLEYNFNSHNVYITADKQKNGIKAYFKIRHNVEDGSVQLADHYQQNTPIGDGPVLLPDNHYLSTQSVL<br>SKDPNEKRDHMVLLEDVTAAGITHGMDELYK**GSGS**LEVLFQGP**GSGS**MISLIAALAVDRVIGMENAMPW<br>NLPADLAWFKRNTLNKPAIMGRHTWESIGRPLPGRKNIILSSQPGTDDRVTWHKSVDEAIAACGDVPEL<br>MVIGGGRVYEQFLPKAQKLYLTHIDAEVEGDTHFPWWWPRIRGPPGS
**FL+28[RNC]**	1-159	MSKGEELFTGVVPILVELDGDVNGHKFSVRGEGEGDATNGKLTLKFICTTGKLPVPWPTLVTTLTYGVL<br>CFSRYPDHMKRHDFFKSAMPEGYVQERTISFKDDGTYKTRAEVKFEGDTLVNRIELKGIDFKEDGNILG<br>HKLEYNFNSHNVYITADKQKNGIKAYFKIRHNVEDGSVQLADHYQQNTPIGDGPVLLPDNHYLSTQSVL<br>SKDPNEKRDHMVLLEDVTAAGITHGMDELYK**GSGS**LEVLFQGP**GSGS**MISLIAALAVDRVIGMENAMPW<br>NLPADLAWFKRNTLNKPVIMGRHTWESIGRPLPGRKNIILSSQPGTDDRVTWVKSVDEAIAACGDVPEI<br>MVIGGGRVYEQFLPKAQKLYLTHIDAEVEGDTHFPDYEPDDWESVFSEFHDADAQNSHSYCFEILERR**G**<br>**SGGDLGSGGYLGSGGLDGSGGVEGSGGFL**WWWPRIRGPPGS
**FL+38[RNC]**	1-159	MSKGEELFTGVVPILVELDGDVNGHKFSVRGEGEGDATNGKLTLKFICTTGKLPVPWPTLVTTLTYGVL<br>CFSRYPDHMKRHDFFKSAMPEGYVQERTISFKDDGTYKTRAEVKFEGDTLVNRIELKGIDFKEDGNILG<br>HKLEYNFNSHNVYITADKQKNGIKAYFKIRHNVEDGSVQLADHYQQNTPIGDGPVLLPDNHYLSTQSVL<br>SKDPNEKRDHMVLLEDVTAAGITHGMDELYK**GSGS**LEVLFQGP**GSGS**MISLIAALAVDRVIGMENAMPW<br>NLPADLAWFKRNTLNKPVIMGRHTWESIGRPLPGRKNIILSSQPGTDDRVTWVKSVDEAIAACGDVPEI<br>MVIGGGRVYEQFLPKAQKLYLTHIDAEVEGDTHFPDYEPDDWESVFSEFHDADAQNSHSYCFEILERRG<br>SGGDLGSGGYLGSGGLDGSGGVEGSGGFLWWWPRIRGPPGS
**FL+58[RNC]**	1-159	MSKGEELFTGVVPILVELDGDVNGHKFSVRGEGEGDATNGKLTLKFICTTGKLPVPWPTLVTTLTYGVL<br>CFSRYPDHMKRHDFFKSAMPEGYVQERTISFKDDGTYKTRAEVKFEGDTLVNRIELKGIDFKEDGNILG<br>HKLEYNFNSHNVYITADKQKNGIKAYFKIRHNVEDGSVQLADHYQQNTPIGDGPVLLPDNHYLSTQSVL<br>SKDPNEKRDHMVLLEDVTAAGITHGMDELYK**GSGS**LEVLFQGP**GSGS**MISLIAALAVDRVIGMENAMPW<br>NLPADLAWFKRNTLNKPVIMGRHTWESIGRPLPGRKNIILSSQPGTDDRVTWVKSVDEAIAACGDVPEI<br>MVIGGGRVYEQFLPKAQKLYLTHIDAEVEGDTHFPDYEPDDWESVFSEFHDADAQNSHSYCFEILERR**G**<br>**SGGDLGSGGYLGSGGLDGSGGVEGSGGFLGSGGLDGSGGLYGSGGVEGS**WWWPRIRGPPGS
**FL+58(GS)[RNC]**	1-159	MSKGEELFTGVVPILVELDGDVNGHKFSVRGEGEGDATNGKLTLKFICTTGKLPVPWPTLVTTLTYGVL<br>CFSRYPDHMKRHDFFKSAMPEGYVQERTISFKDDGTYKTRAEVKFEGDTLVNRIELKGIDFKEDGNILG<br>HKLEYNFNSHNVYITADKQKNGIKAYFKIRHNVEDGSVQLADHYQQNTPIGDGPVLLPDNHYLSTQSVL<br>SKDPNEKRDHMVLLEDVTAAGITHGMDELYK**GSGS**LEVLFQGP**GSGS**MISLIAALAVDRVIGMENAMPW<br>NLPADLAWFKRNTLNKPVIMGRHTWESIGRPLPGRKNIILSSQPGTDDRVTWVKSVDEAIAACGDVPEI<br>MVIGGGRVYEQFLPKAQKLYLTHIDAEVEGDTHFPDYEPDDWESVFSEFHDADAQNSHSYCFEILERR**G**<br>**SGSGSGSGSGSGSGSGSGSGSGSGSGSGSGSGSGSGSGSGSGSGSGSGS**WWWPRIRGPPGS

Supplementary Table 1. DHFR RNC sequences.

The N-terminal muGFP (green), functioning as an affinity tag, precedes a 3C cleavage sequence (blue) that is flanked on either side by $(GS)_2$ linkers (bold). The subsequent DHFR sequence fragment is followed by a C-terminal stall-inducing sequence derived from *M. succiniciproducens* SecM[41]. Structural analysis of ribosomes stalled on wild-type *E. coli* SecM indicates that various stalling scenarios are possible[65]. When describing our RNCs we assume that the ribosome stalls at the first available opportunity, with the penultimate $tRNA^{Pro}$ in the A-site and the first 8 residues from the stall-inducing sequence occupying the exit tunnel. In the full-length RNCs an additional disordered linker precedes the stall sequence (bold). In 1-37[RNC], the GS linker following the 3C cleavage sequence is extended to improve capture by the GFP affinity resin. Sites of point mutations are underlined.
