# Supplementary figures and images for: Resolving chaperone-assisted protein folding on the ribosome at the peptide level
Source: Nat Struct Mol Biol. 2024 Jul 10;31(12):1888–97. doi: 10.1038/s41594-024-01355-x (PMC11638072; doi:10.1038/s41594-024-01355-x)

Source data for Extended data Fig. 1 - unprocessed gels

Panel 1b

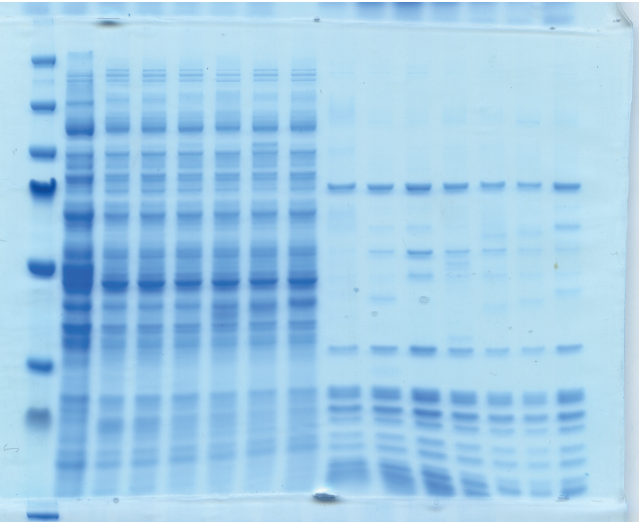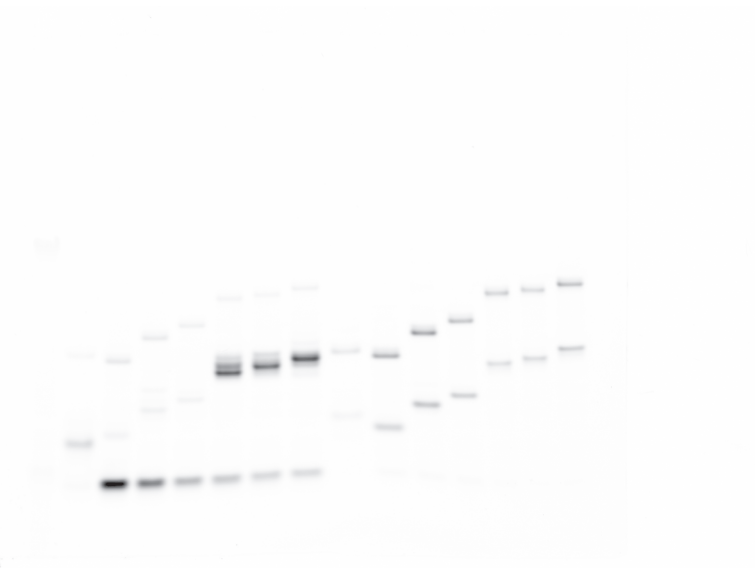

Panel 1c

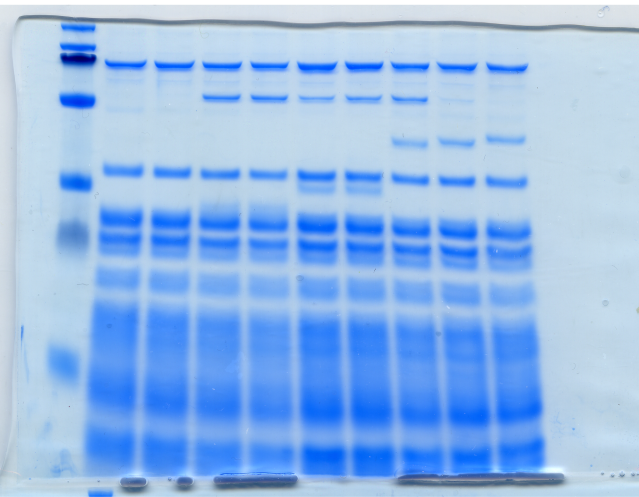

Panel 1d

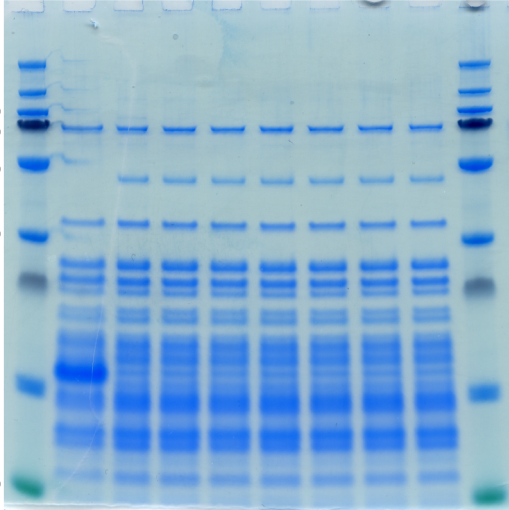

Panel 1f

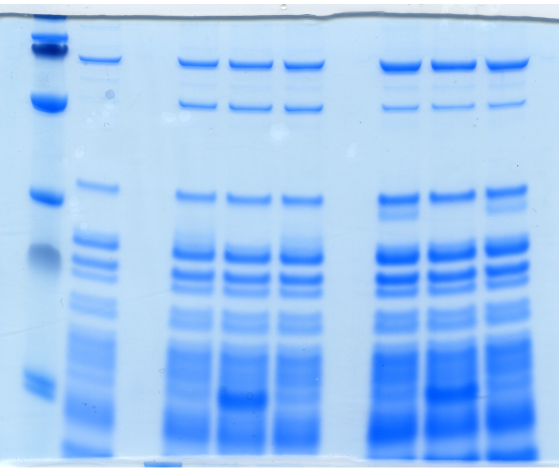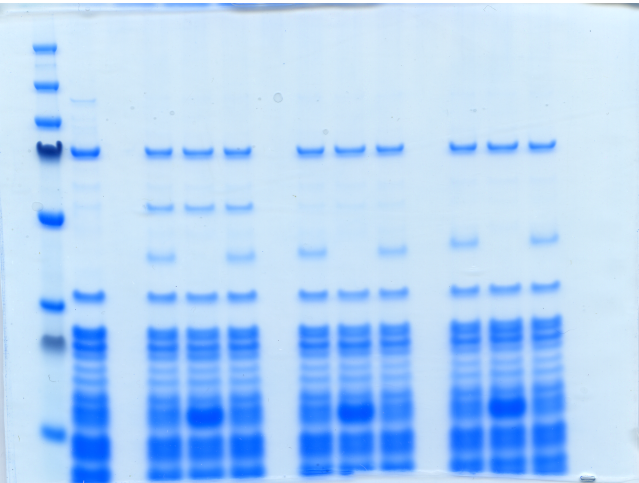

Supplement: Supplementary file 6 — Unprocessed gels. [file 41594_2024_1355_MOESM6_ESM.pdf]

Source data for Extended data Fig. 2 - unprocessed western blots

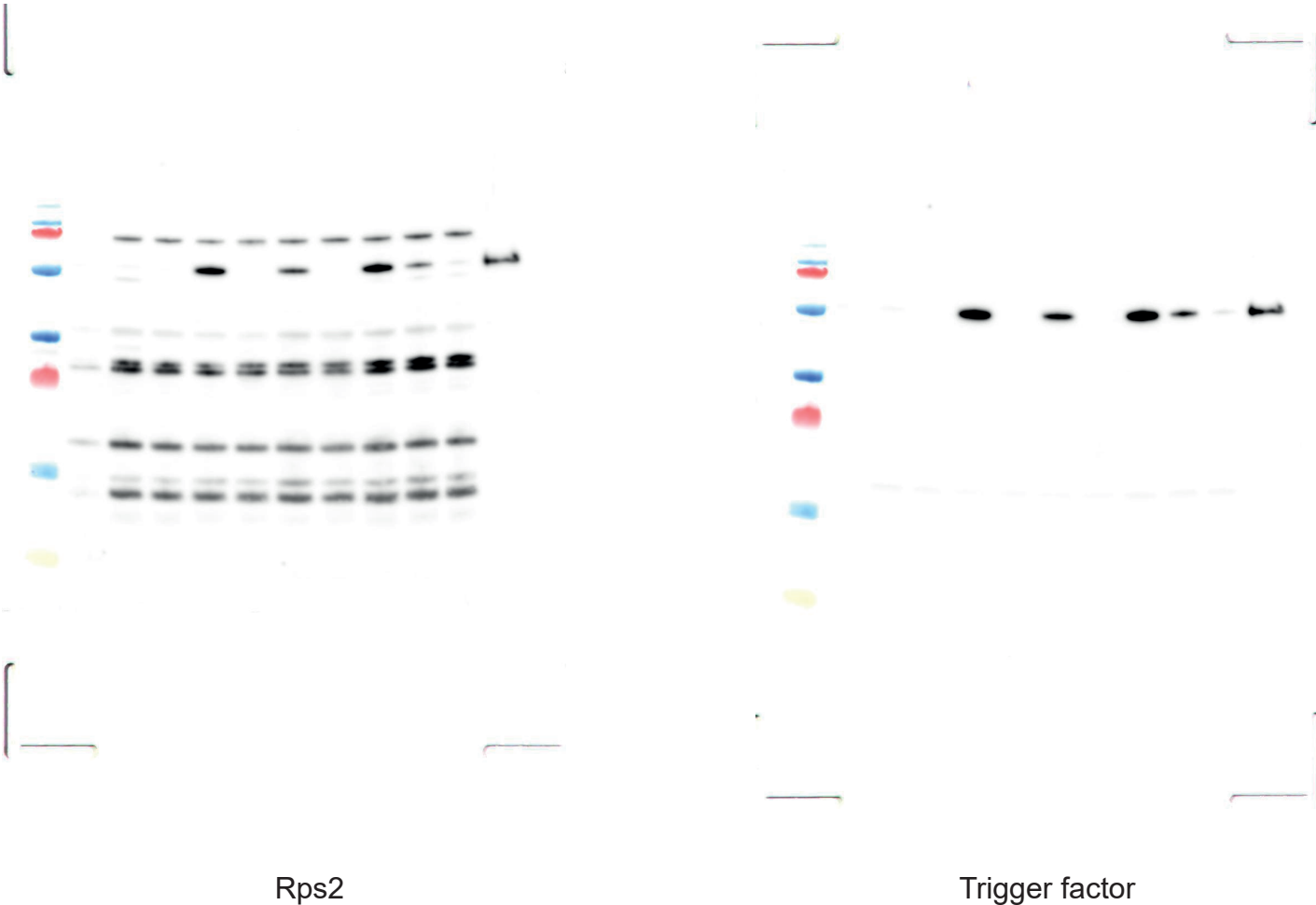

Supplement: Supplementary file 8 — Unprocessed gels. [file 41594_2024_1355_MOESM8_ESM.pdf]

Source data for Extended data Fig. 6f

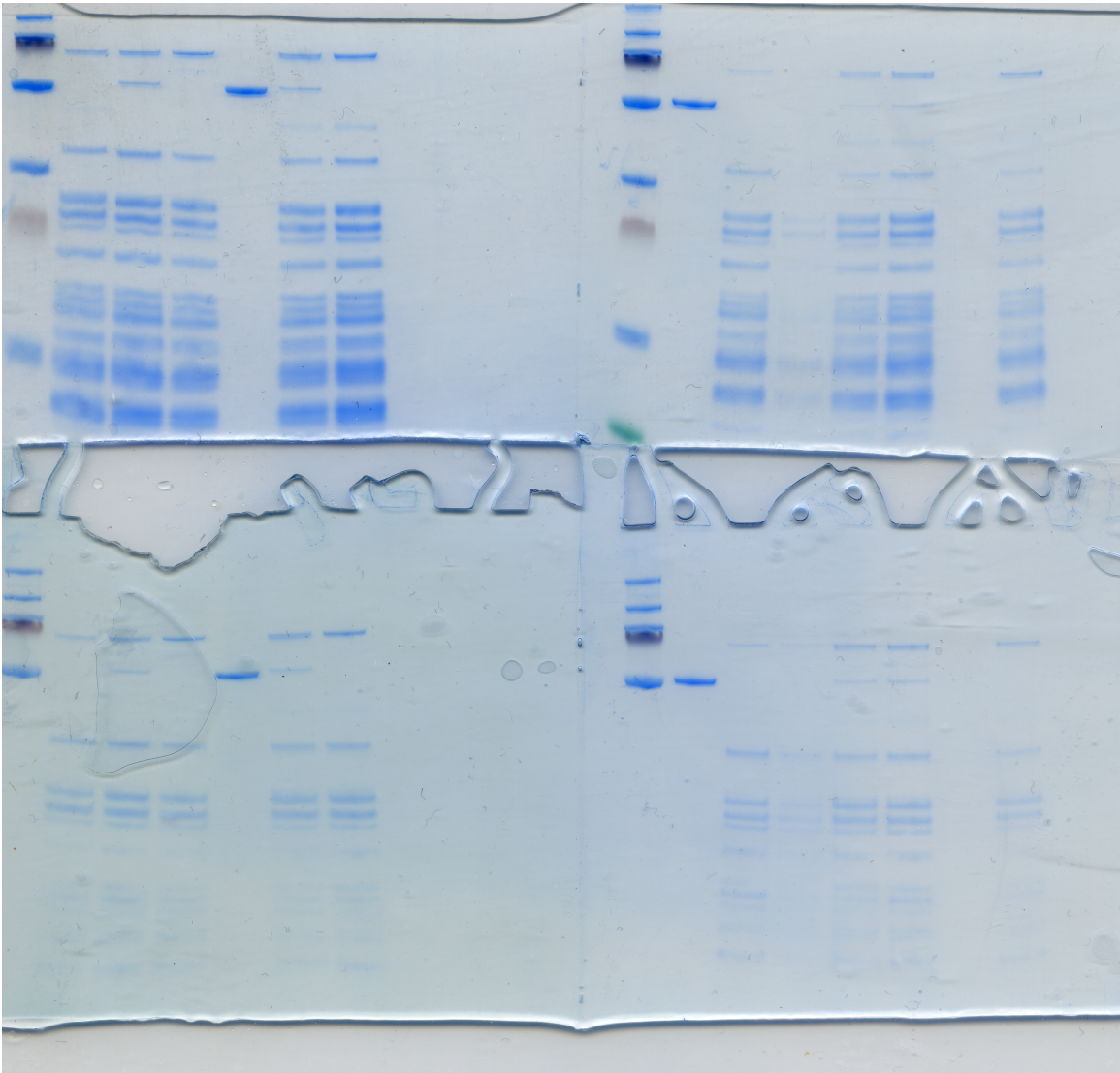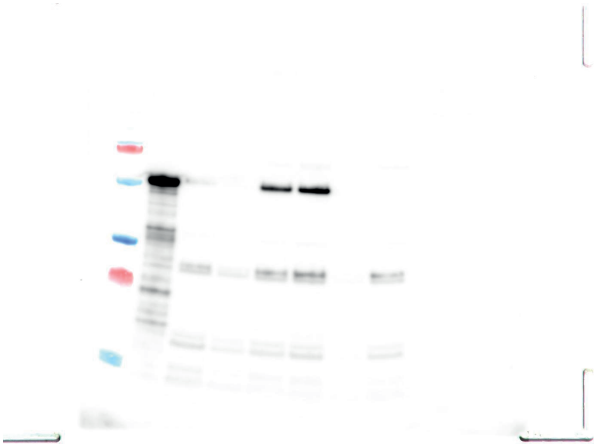

Supplement: Supplementary file 9 — Unprocessed gels. [file 41594_2024_1355_MOESM9_ESM.pdf]

Source data for Extended data Fig. 8a

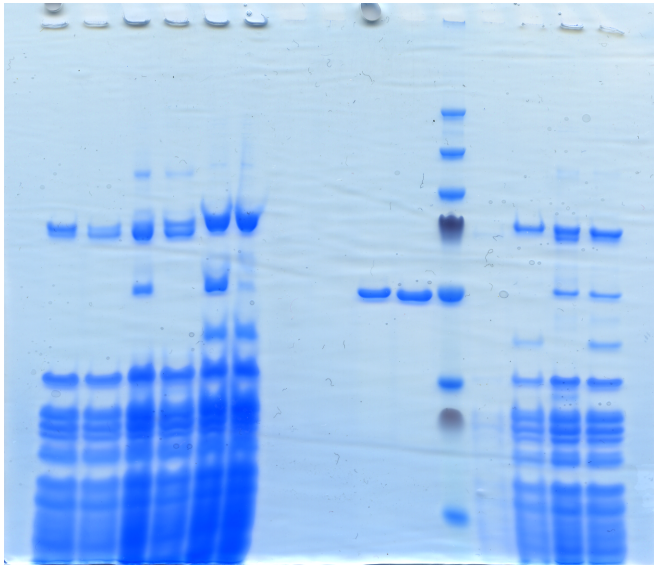

Supplement: Supplementary file 11 — Unprocessed gels. [file 41594_2024_1355_MOESM11_ESM.pdf]
